# Production, characterization and bioinformatics analysis of l-asparaginase from a new *Stenotrophomonas maltophilia* EMCC2297 soil isolate

**DOI:** 10.1186/s13568-020-01005-7

**Published:** 2020-04-15

**Authors:** Nada A. Abdelrazek, Walid F. Elkhatib, Marwa M. Raafat, Mohammad M. Aboulwafa

**Affiliations:** 1 Department of Microbiology and Immunology, Faculty of Pharmaceutical Sciences and Pharmaceutical Industries, Future University in Egypt, Cairo, Egypt; 2 Department of Microbiology and Immunology, School of Pharmacy & Pharmaceutical Industries, Badr University in Cairo (BUC), Entertainment Area, Badr City, Cairo 11829 Egypt; 3 Department of Microbiology and Immunology, Faculty of Pharmacy, Ain Shams University, African Union Organization St., Abbassia, Cairo 11566, Egypt

**Keywords:** l-asparaginase, *Stenotrophomonas maltophilia*, Production optimization, Characterization, Response Surface Methodology

## Abstract

An exhaustive screening program was applied for scoring a promising l-asparaginase producing-isolate. The recovered isolate was identified biochemically and molecularly and its l-asparaginase productivity was optimized experimentally and by Response Surface Methodology. The produced enzyme was characterized experimentally for its catalytic properties and by bioinformatics analysis for its immunogenicity. The promising l-asparaginase producing-isolate was selected from 722 recovered isolates and identified as *Stenotrophomonas maltophilia* and deposited at Microbiological Resources Centre (Cairo Mircen) under the code EMCC2297. This isolate produces both intracellular (type I) and extracellular (type II) l-asparaginases with about 4.7 fold higher extracellular l-asparaginase productivity. Bioinformatics analysis revealed clustering of *Stenotrophomonas maltophilia*l-asparaginase with those of *Pseudomonas* species and considerable closeness to the two commercially available l-asparaginases of *E. coli* and *Erwinia chrysanthemi*. Fourteen antigenic regions are predicted for *Stenotrophomonas maltophilia*l-asparaginase versus 16 and 18 antigenic regions for the *Erwinia chrysanthemi* and *E. coli*l-asparaginases. Type II l-asparaginase productivity of the test isolate reached 4.7 IU/ml/h and exhibited maximum activity with no metal ion requirement at 37 °C, pH 8.6, 40 mM asparagine concentration and could tolerate NaCl concentration up to 500 mM and retain residual activity of 55% at 70 °C after half an hour treatment period. Application both of random mutation by gamma irradiation and Response Surface Methodology that determined 38.11 °C, 6.89 pH, 19.85 h and 179.15 rpm as optimum process parameters could improve the isolate l-asparaginase productivity. Maximum production of about 8 IU/ml/h was obtained with 0.4% dextrose, 0.1% yeast extract and 10 mM magnesium sulphate. In conclusion l-asparaginase of the recovered *Stenotrophomonas maltophilia* EMCC2297 isolate has characters enabling it to be used for medical therapeutic application.

## Introduction

l-asparaginase is a therapeutic enzyme used for the treatment of acute lymphocytic leukemia (Sinha et al. 2013). This enzyme is present in many bacteria and plants but not in mankind and it has a crucial role in storage and transport of nitrogen required for protein biosynthesis. l-asparaginase is synthesized at a slow rate in malignant cells as a result of their reduced capability to synthesize l-asparagine synthetase as compared to normal cells. Thus applying l-asparaginase to tumor cells can deplete its content of l-asparagine rendering them unable to synthesize protein as well as RNA and DNA and inducing apoptosis in these cells (Bansal et al. 2012). l-asparaginase is used in food industry to prevent the formation of acrylamide (carcinogenic toxicant) (Krishnapura et al. 2016) in food products manufactured at temperatures exceeding 100 °C. Additionally, the amount of asparagine in leukemic patients and food materials can be detected by this enzyme (Batool et al. 2016). Microbial l-asparaginases are marketed for therapeutic applications under the brand names Kidrolase^®^, Elspar^®^ and for food applications under the commercial name Acrylaway^®^ and PreventASe^®^ (Krishnapura et al. 2016). In this article we succeeded to recover a bacterial soil isolate, *Stenotrophomonas maltophilia* EMCC2297, with promising l-asparaginase productivity. As revealed by bioinformatics analysis, the l-asparaginase of this isolate is less immunogenic than those of *E. coli* and *Erwinia chrysanthemi*, the commercially available ones. In this study, *Stenotrophomonas maltophilia* isolate recovered in this study was improved by gamma mutation and the production of the resultant variant of l-asparaginase was optimized by RSM experimental design and laboratory experiments to be introduced as a candidate for l-asparaginase of medical and pharmaceutical use as antileukemic agent.

## Materials and methods

### Chemicals

Unless otherwise stated the chemicals applied in this study were obtained from, (ADWIC) chemicals, Abuzaabal, Egypt. The source of l-asparagine monohydrate was from AppliChem GmbH, Darmstadt, Germany.

### Isolate recovery from soil sample and qualitative assessment of l-asparaginase production

Screening of 722 recovered isolates from different soil samples resulted in the scoring of an isolate with promising production capability for l-asparaginase as assessed qualitatively using the method described by Izadpanah et al. (2014). The applied method relied on the detection of pink zone surrounding l-asparaginase bacterial producer colonies on modified M9 agar medium containing 1% w/v asparagine and phenol red as indicator.

### Preparation of inoculum, production and quantitative assay of l-asparaginase by the selected recovered isolate

Both inoculum preparation and l-asparaginase production by the test isolate were carried out in 250 ml Erlenmeyer flasks containing M9 with 1% w/v asparagine (20 ml in case of inoculum preparation and 50 ml in case of l-asparaginase production condition). In both cases incubation was carried out at 37 °C and 180 rpm for 24 h. The inoculum size used in the production condition was 2% v/v from adjusted growth at O.D_600nm_ of 1.0 (Mahajan et al. 2012). Quantitative assay of l-asparaginase production was determined for the extracellular type II l-asparaginase obtained in the supernatant resulted from centrifugation of the culture broth at 1957×*g* and 4 °C for 20 min (Jain et al. 2012). While the intracellular type I enzyme was assayed in the lysate produced by sonicating the washed and resuspended pellets (in 50 mM Tris HCl pH 7.5) in 30 ml lysis buffer (Straight et al. 2007). The resulting lysate was centrifuged for 15 min at 17,609×*g* and 4 °C for the removal of unbroken cells and cellular debris (Sakr et al. 2014). The intracellular enzyme activity was then determined in the collected supernatant. l-asparaginase quantitative assessment was carried out using the method described by Mashburn and Wriston (1963). The enzyme acts on l-asparagine substrate contained in the assay to release ammonia. One unit of l-asparaginase activity was defined as the amount of the enzyme that required for the release of 1 µmole ammonia under the assay condition (37 °C, pH 8.6, 1 h incubation period) (Mahajan et al. 2014).

### Identification of the selected test isolate

The selected isolate of the highest l-asparaginase productivity was characterized by investigation under the microscope, determining its biochemical profile by Vitek^®^ system and its identity was confirmed by sequencing the 16S rRNA gene of the organism chromosomal DNA.

### Bioinformatics analysis

The relatedness of l-asparaginase from *Stenotrophomonas maltophilia* to those retrieved from NCBI database including those from *E. coli* and *Erwinia chrysanthemi* (commercially available as Elspar and Erwinaze, respectively) were inferred by the Maximum Likelihood method based on the JTT matrix-based model (Jones et al. 1992). The molecular evolutionary genetic analyses were done using MEGA X (Kumar et al. 2018). The antigenic sites in *Stenotrophomonas maltophilia*l-asparaginase relative to those in the commercially available l-asparaginases of *E. coli* and *Erwinia chrysanthemi* could be predicted by the software developed using the method of Kolaskar and Tongaonkar (1990) (EMBOSS: antigenic—Bioinformatics web site http://www.bioinformatics.nl/cgi-bin/emboss/antigenic).

### Characterization of *Stenotrophomonas maltophilia* EMCC2297 l-asparaginase

The enzyme of the test isolate (cell free culture supernatant) was tested for various characters that will determine its application scope. These included stability at different temperatures, optimum temperature and pH for activity, activity at different concentrations each of sodium chloride, asparagine (substrate) and metal salts.

### Isolate improvement for l-asparaginase production

Treatment with gamma radiation was used for improving l-asparaginase production by the test isolate. Aliquots (5 ml each) of an overnight growth culture of the test isolate, in a glass tubes were treated at various gamma rays doses (from 0.1 to 5 KGy) (Hoe et al. 2016). After treatment the l-asparaginase productivity for a number of randomly selected colonies were assessed qualitatively as well as quantitatively (Abdelrazek et al. 2019) and compared to the enzyme productivity of the parent isolate. The variant that gave the maximum enzyme production was subjected for further studies.

### Optimization of l-asparaginase production by the selected variant

l-asparaginase production by the selected variant was tested at different temperatures, initial pH values, periods of incubation, agitation levels and various components of the used culture media. For each tested parameters the enzyme productivity was measured at the end of incubation period as described by Abdelrazek et al. (2019). While a time course for enzyme production was carried out for determining the incubation period required for maximum l-asparaginase production.

### Optimization by Response Surface Methodology

From a number of experiments carried out one at a time, four parameters affecting l-asparaginase production which included temperature of incubation, designated as (A); initial pH, designated as (B); incubation period, designated as (C) and rate of agitation designated as (D) were subjected to analysis by Response Surface Methodology experimental design (Box–Behnken design). Three levels of each test parameter were applied to describe l-asparaginase production. The mean level, designated as (0) which represents the one of the maximum l-asparaginase production, while the other 2 levels represent the one above, designated as (+1) and the one below, designated as (− 1) this mean level. The range of variables studied is represented as Additional file 1: Table S7. The analyses procedures were carried out as described by Abdelrazek et al. (2019) using the values listed in Additional file 1: Table S7.

### Optimization by adjusting the components of the used culture media

The effect of various sources of carbon and nitrogen as well as metal ions was tested for studying their effect on enzyme production by the test variant. Dextrose, yeast extract and sulphate salt of magnesium as a carbon, nitrogen and metal ion sources, respectively were examined at various concentrations.

### Statistical data analyses and graphical representations

The mean values and standard deviation (presented as error bars) from triplicate conducted experiments were determined. Both one way ANOVA and Dunnett’s Multiple Comparison Test were applied using Graph Pad Prism Version 5.0 software for data analyses.

## Results

### Isolation and identification of a *Stenotrophomonas maltophilia* isolate

A soil isolate with potential production of l-asparaginase could be scored as mentioned in “Materials and methods” among 722 recovered isolates. Both biochemical reaction using Vitek^®^ system and 16S rRNA sequencing revealed the identity of this organism as *Stenotrophomonas maltophilia*. This isolate was deposited at Microbiological Resources Centre (Cairo Mircen) under the code EMCC2297 and its 16S rRNA sequence was submitted and deposited in NCBI database under the Accession code MG665996. The test isolate used in the present study could produce l-asparaginase extracellularly (Type II l-asparaginase) at a level 4.7 folds more than its intracellular level.

### Phylogeny and antigenicity for l-asparaginase of *Stenotrophomonas maltophilia*

**Fig. 1:**
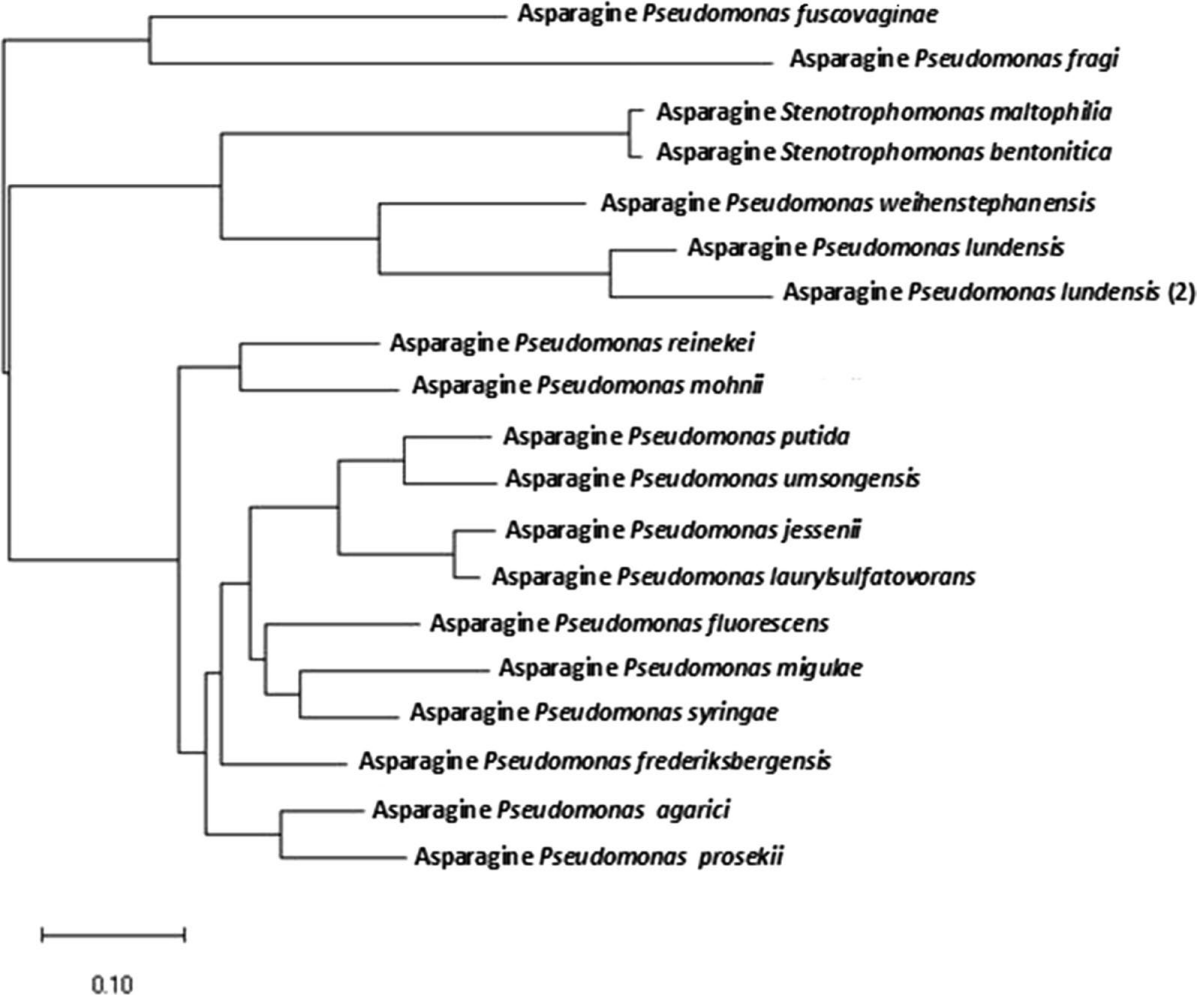
Phylogenetic analysis of l-asparaginase produced by *Stenotrophomonas maltophilia* as determined by Maximum Likelihood method. Non redundant amino acid sequences of l-asparaginases retrieved from NCBI databases with similarities more than 56% were aligned against the query sequence (l-asparaginase of *Stenotrophomonas maltophilia*) and used for the tree construction. Additional file 1: Table S1 shows query coverage, E value, percent identity and accession numbers of amino acid sequences of l-asparaginases for the tested bacterial species as revealed by NCBI databases

**Fig. 2:**
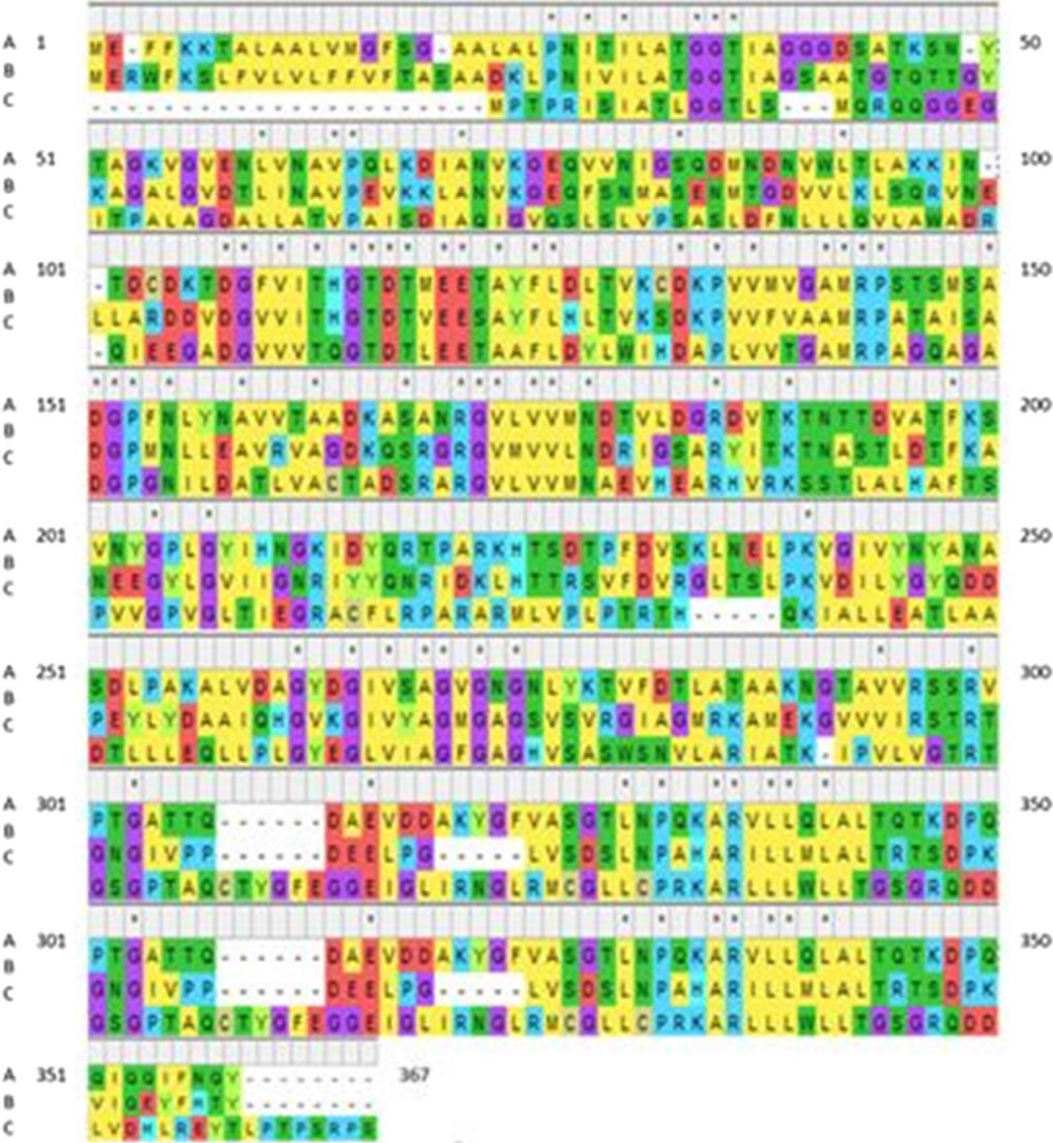
Amino acid sequences alignment of l-asparaginase of *Stenotrophomonas maltophilia* (**c**) relative to that of *E. coli* B354 (**a**) and *Erwinia chrysanthemi* (**b**)

Figure 1 shows the tree describing the molecular phylogeny of l-asparaginase produced by *Stenotrophomonas maltophilia* test isolate. This tree was produced upon aligning amino acids sequence of *Stenotrophomonas maltophilia*l-asparaginase against the corresponding sequences deposited in NCBI database of similarities not less than 58%. While, Fig. 2 shows the amino acid alignment of the target query l-asparaginase against those of the two commercially available *E. coli* and *Erwinia chrysanthemi*l-asparaginases (Elspar and Erwinaze, respectively). For comparison of plant type l-asparaginase and bacterial species ones, a molecular phylogenetic tree for *Stenotrophomonas maltophilia*l-asparaginase and those of the two reference bacterial strains, *E. coli* and *Erwinia chrysanthemi,* as well as the plant type of *Medicago truncatula* was constructed (Additional file 1: Fig. S1). Pairwise distances among bacterial l-asparaginases illustrated in Fig. 1 are shown in Additional file 1: Table S3 while the corresponding distances among bacterial species l-asparaginases and those of plant type ones in Additional file 1: Fig. S2 are illustrated in Additional file 1: Table S3.

Analysis by EMBOSS antigenic explorer^®^ (Rice et al. 2000) revealed 18, 16 and 14 antigenic regions, for *E. coli*, *Erwinia chrysanthemi* and *Stenotrophomonas maltophilia*l-asparaginases, respectively. The positions of these antigenic regions together with their amino acid sequences are shown in Table 1.

**Table 1:** Antigenic regions, their positions and sequences of *E. coli*, *Erwinia chrysanthemi* and *Stenotrophomonas maltophilia*l-asparaginases

*E. coli*l-asparaginase (348 aa)^a^				*Erwinia chrysanthemi*l-asparaginase^b^ (348 aa)				*Stenotrophomonas maltophilia*l-asparaginase^c^ (335 aa)			
Anti-genic region	Start	End	Sequence	Anti-genic region	Start	End	Sequence	Anti-genic region	Start	End	Sequence
1	323	333	KARVLLQLALT	1	6	20	KSLFVLVLFFVFTS	1	27	73	TPALAGDALLATVPAISDIAQIGVQSLSLVPSASLDFNLLLQVLAWA
2	224	243	PFDVSKLNELPKVGIVYNYA	2	121	142	ESAYFLHLTVKSDKPVVFVAAM	2	155	183	VHEARHVRKSSTALHAFTSPVVGPVLT
3	245	257	ASDLPAKALVDG	3	88	101	GDVVLKLSQRVL	3	145	153	RGVLVVMNA
4	118	135	AYFLDLTVKCDKPVVMVG	4	290	297	KGVVVIRS	4	294	312	MCGLLCPRKARLLLWLLTG
5	6	16	KTALAALVMGF	5	156	165	LLEAVRVAGD	5	208	239	QKIALLEATLAADTLLLEQLLPLGYEGLVIAG
6	165	174	NRGVLVVMND	6	311	333	LPGLVSDSLNPAHARILLMLALT	6	96	114	TAAFLDYLWIHDAPLVVTG
7	49	70	AGKVGVENLVNAVPQLKDIANV	7	22	34	ADKLPNIVILATG	7	242	266	AGHVSASWSNVLARIATKIPVLVGT
8	151	159	LYNAVVTAA	8	172	179	GVMVVLND	8	132	141	DATLVACTAD
9	286	298	GTAVVRSSRVPTG	9	107	114	VDGVVITH	9	82	88	DGVVVTQ
10	72	79	EQVVNIG	10	251	270	PEYLYDAAIQHGVKGIVYAG	10	186	194	GRACFLRPA
11	190	204	VATFKSVNYGPLGYI	11	226	248	TRSVFDVRGLTSLPKVDILYGYQ	11	196	205	ARMLVPLPTR
12	259	265	DGIVSAG	12	205	214	YLGVIIGNRI	12	317	331	DDLVDHLREYTLPP
13	88	94	WLTLAKK	13	52	73	AGALGVDTLINAVPEVKKLANV	13	273	279	TAQCTYG
14	18	32	GAALALPNITILAG	14	337	345	DPKVIQEYF	14	5	13	RISIATLGG
15	272	282	YKTVFDTLATA	15	274	281	GSVSVRGI				
16	311	317	YGFVASG	16	303	309	GIVPPDE				
17	339	345	QQIQQIF								
18	104	109	GFVITH								

^a^*E. coli* FDA approved l-asparaginase (marketed under the brand name Elspar)

^b^*Erwinia chrysanthemi* (*Dickeya chrysanthemi* or *Pectobacterium chrysanthemi*) l-asparaginase (marketed under the brand name Erwinaze)

^c^*Stenotrophomonas maltophilia (Pseudomonas maltophilia* or *Xanthomonas maltophilia)*

### Characterization of *Stenotrophomonas maltophilia* EMCC2297 l-asparaginase

**Fig. 3:**
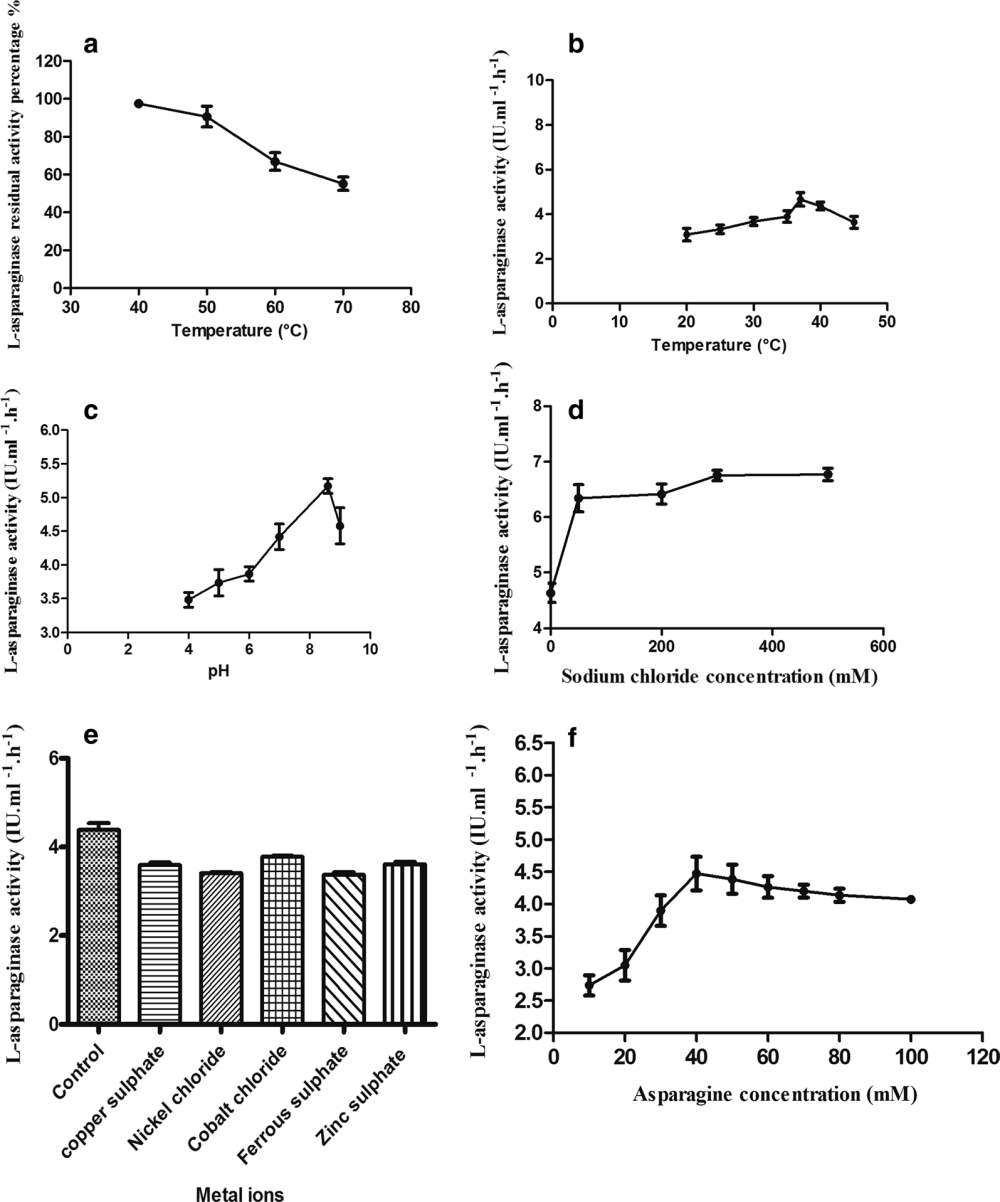
Stability to temperature treatment (**a**), activities at various temperatures (**b**), pHs (**c**) and sodium chloride concentrations (**d**), substrate concentrations (**e**) and metal ions (**f**) of l-asparaginase produced by *Stenotrophomonas maltophilia* EMCC2297. In all cases (**a**–**d**) p-values were < 0.0001 which indicates high significance of the correlation

Thermal stability study showed that l-asparaginase of the test isolate could tolerate temperature treatment up to 50 °C for 30 min treatment period without appreciable reduction in its activity. The maximum activity was attained at 37 °C, pH 8.6 and 40 mM asparagine concentration and there was an increase in the activity by about 50% at half molar sodium chloride concentration. The results of the present study for *Stenotrophomonas maltophilia*l-asparaginase showed that the chloride salts of either nickel or cobalt and the sulphate salts of either copper or ferrous were not required for the enzyme activity (Fig. 3).

### *Stenotrophomonas maltophilia* EMCC2297 improvement for l-asparaginase production

Random mutation by treatment with gamma rays (5 KGy) could successfully improve the test isolate for l-asparaginase production. The selected variant showed 1.6 folds increase in l-asparaginase productivity compared to the wild type parent strain.

### Production optimization of l-asparaginase by *Stenotrophomonas maltophilia* variant

#### (a) Optimization through studying environmental conditions and statistical design of experiments by Response Surface Methodology (RSM)

**Fig. 4:**
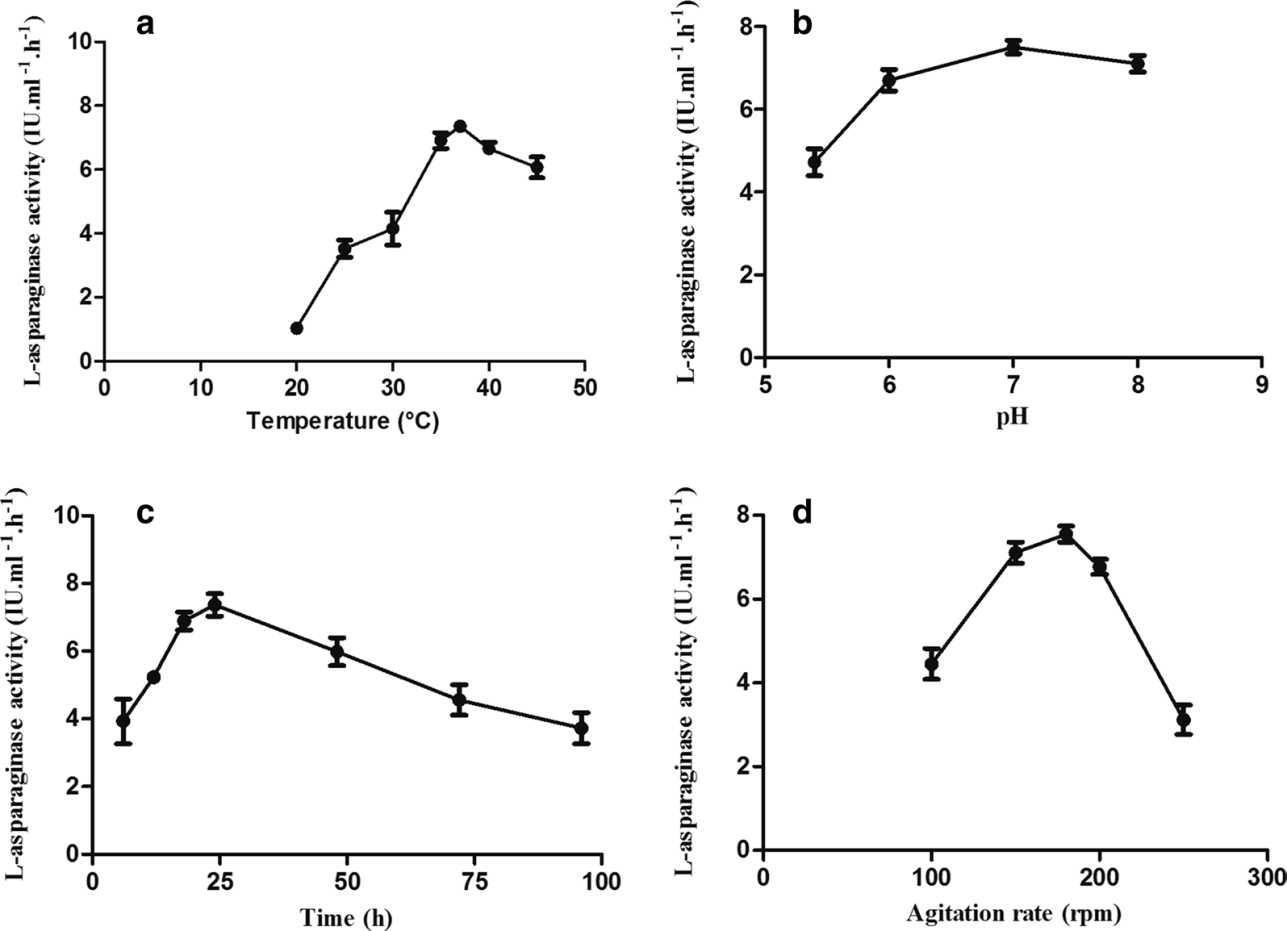
l-asparaginase production by *Stenotrophomonas maltophilia* EMCC2297 variant at different temperatures of incubation (**a**), initial pH values (**b**), incubation periods (**c**) and rates of agitation (**d**)

The preliminary studies (Fig. 4) revealed that; the used *Stenotrophomonas maltophilia* variant could maximally produce l-asparaginase at 37 °C. Regarding the effect of pH on l-asparaginase production by the test variant, it was found that maximum enzyme productivity occurred at initial pH 7. Maximum l-asparaginase production occurred at 24 h of incubation. Regarding agitation, low rates did not support high enzyme production, while increasing agitation rate was accompanied by gradual increase in l-asparaginase production till maximum level that was achieved at 180 rpm, followed by a decline.

The four previous tested environmental conditions (temperature of incubation, initially used pH, incubation period and the rate of agitation) were subjected to RSM model for designing a number of experiments that could optimally increase l-asparaginase production. Table 2 shows the model results (observed, predicted and residual values). The square root values of l-asparaginase productivity in relation to the tested parameters (variables) could be described by polynomial equation of second order as follows:

$$\begin{aligned} {\text{Sqrt}}\left( {\text{L-asparaginase activity}} \right) & = + 2.72 + 0.018*{\text{A}}{-}2.110{\text{E}} - 003*{\text{B}} - 0.070*{\text{C}} \\ & \quad {-}7.744{\text{E}} - 003*{\text{D}} - 0.058*{\text{A}}*{\text{B}} - 0.024*{\text{A}}*{\text{C}} \\ & \quad + 0.085*{\text{A}}*{\text{D}} + 0.0 1 7*{\text{B}}*{\text{C }} + \, 0.0 3 1*{\text{B}}*{\text{D}} \\ & \quad - 0.0 3 2*{\text{C}}*{\text{D}} - 0. 9 2*{\text{A}}^{ 2} - 0. 8 8*{\text{B}}^{ 2} + 0.0 30*{\text{C}}^{ 2} - 0.0 6 4*{\text{D}}^{ 2} \\ \end{aligned}$$.

**Table 2:** Optimization of process parameters for l-asparaginase production by *Stenotrophomonas maltophilia* EMCC2297 variant using Box–Behnken design

Experiment	Temp (°C)	pH	Time (h)	Agitation (rpm)	Square root of l-asparaginase activity (IU/ml/h)		Residual value
					Observed value	Predicted value	
1	35	6	33	175	2.5	2.47	0.03
2	40	6	33	175	2.6	2.62	− 0.02
3	35	8	33	175	2.62	2.58	0.04
4	40	8	33	175	2.48	2.5	− 0.02
5	37.5	7	18	150	2.75	2.74	0.01
6	37.5	7	48	150	2.67	2.66	0.01
7	37.5	7	18	200	2.79	2.78	0.01
8	37.5	7	48	200	2.58	2.58	0
9	35	7	33	150	2.62	2.64	− 0.02
10	40	7	33	150	2.55	2.51	0.04
11	35	7	33	200	2.44	2.46	− 0.02
12	40	7	33	200	2.7	2.66	0.04
13	37.5	6	18	175	2.75	2.76	− 0.01
14	37.5	8	18	175	2.71	2.72	− 0.01
15	37.5	6	48	175	2.6	2.58	0.02
16	37.5	8	48	175	2.64	2.61	0.03
17	35	7	18	175	2.69	2.69	0
18	40	7	18	175	2.77	2.77	0
19	35	7	48	175	2.57	2.6	− 0.03
20	40	7	48	175	2.56	2.59	− 0.03
21	37.5	6	33	150	2.6	2.61	− 0.01
22	37.5	8	33	150	2.52	2.55	− 0.03
23	37.5	6	33	200	2.53	2.54	− 0.01
24	37.5	8	33	200	2.58	2.59	− 0.01
25^a^	37.5	7	33	175	2.71	2.72	− 0.01
26^a^	37.5	7	33	175	2.73	2.72	0.01
27^a^	37.5	7	33	175	2.74	2.72	0.02

^a^Refers to the experiments that were carried out in triplicates at the mean value of each tested parameter (temperature of incubation, initial pH, time of incubation, rate of agitation)

The letters A, B, C and D refer to temperature of incubation, initially used pH, incubation period and rate of agitation, respectively while the l-asparaginase activity was determined in terms of square root values.

ANOVA of the resultant quadratic design was shown as Additional file 1: Table S3 online. For the Model obtained F-value (15.47), a p-value lower than 0.0001. The R^2^ was computed to be 0.9475, The “Adjusted R-Squared” of 0.8863, “Predicted R-Squared” of 0.7028. “Adequate precision” was 13.564. The coefficient of variation value was 1.23%. The above mentioned criteria and considerations are used as indicators for the regression model to be valid and adequate. In our study the parameters that confirmed to be significance for l-asparaginase production by the test variant are mentioned in Table 3.

**Table 3:** Summarization of parameters that significantly influence l-asparaginase production by *Stenotrophomonas maltophilia* EMCC2297 variant arranged in a descending order

Order	Process parameter factor
1	Time of incubation
2	Temperature of incubation in square terms
3	Initial pH in square terms
4	Interaction between incubation temperature and agitation rate
5	Square term of agitation rate
6	Interaction between incubation temperature and initial pH

### Bidirectional cross interactions of the tested parameters for l-asparaginase production by the used *Stenotrophomonas maltophilia* EMCC2297 variant

**Fig. 5:**
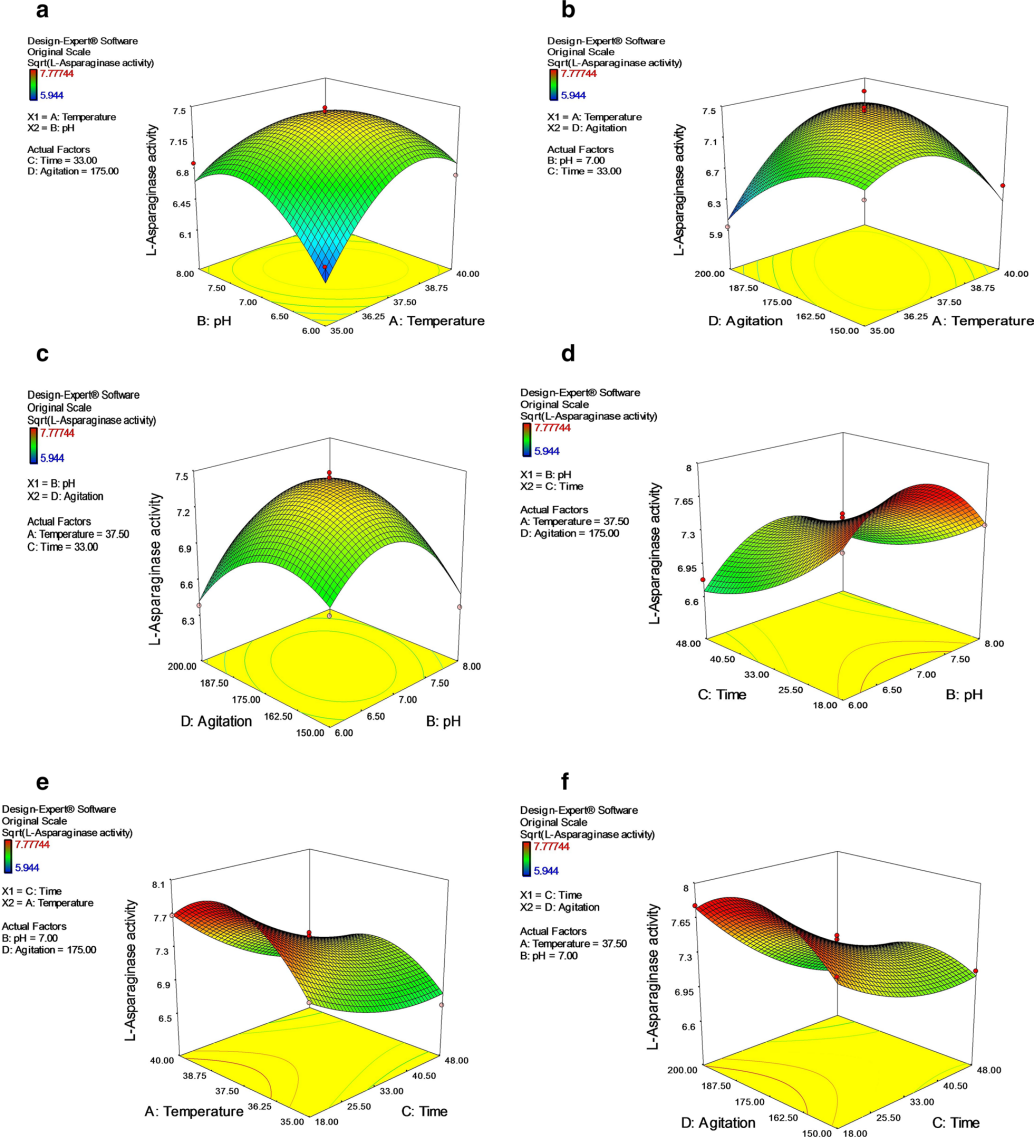
Graphs showing process parameters optimization for l-asparaginase production by *Stenotrophomonas maltophilia* EMCC2297 variant as revealed by Response surface methodology analysis. The graphs show the interactions between: **a** temperature of incubation (°C) and pH; **b** temperature of incubation (°C) and rate of agitation (rpm); **c** pH and rate of agitation (rpm); **d** pH and incubation periods (h); **e** incubation periods (h) and temperature of incubation (°C); and **f** incubation periods (h) and rate of agitation (rpm)

The bidirectional cross interactions were expressed as 3D and 2D for response surface and contour plots, respectively (Fig. 5). These plots were constructed from the data obtained from the regression equation. The effect of interaction of temperature of incubation with pH and rate of agitation are shown in Fig. 5a, b. These interactions resulted in predicted values for maximum productivity of l-asparaginase of 7.42 IU/ml/h at pH 6.87 and temperature of incubation 37.68 °C and of 7.43 IU/ml/h at the same temperature of incubation (37.68 °C) and rate of agitation 175.47 rpm. The effect of interaction of pH with the rate of agitation and incubation period is shown in Fig. 5c, d.

These interactions resulted in predicted values for maximum productivity of l-asparaginase of 7.43 IU/ml/h at pH 7 and 173 rpm and of 7.98 IU/ml/h at the same pH (7) and at an incubation period of 18.0 h. The effect of interaction of incubation period with temperature of incubation and rate of agitation are shown in Fig. 5e, f. These interactions resulted in predicted values for maximum productivity of l-asparaginase of 7.98 IU/ml/h at temperature of incubation at 37.5 °C and incubation period 18.0 h and of 7.98 IU/ml/h at the same incubation period (18.0 h) and at 175 rpm rate of agitation. These graphs can lead us to some conclusions regarding the enzyme production; decreasing the pH of the medium from 7 increases the l-asparaginase productivity. This effect is much more pronounced when the temperature is shifted away from 37 °C, when the agitation increased above 175 or decreasing incubation time. Increasing the rate of agitation increases the production of l-asparaginase, this effect gets much more significant by increasing temperature or decreasing time towards 18 h.

The main goal of Response Surface Methodology application is to determine the process value for each variable to be optimum for maximizing the output of the studied process. In this study application of the model resulted in prediction of maximum production level of l-asparaginase by the used *Stenotrophomonas maltophilia* variant of 7.95 IU/ml/h. This maximum enzyme level can be obtained at 38.11 °C temperature of incubation, pH of 6.89, 19.85 h incubation period and 179.15 rpm rate of agitation.

#### (b) By testing the components of the applied culture medium

**Fig. 6:**
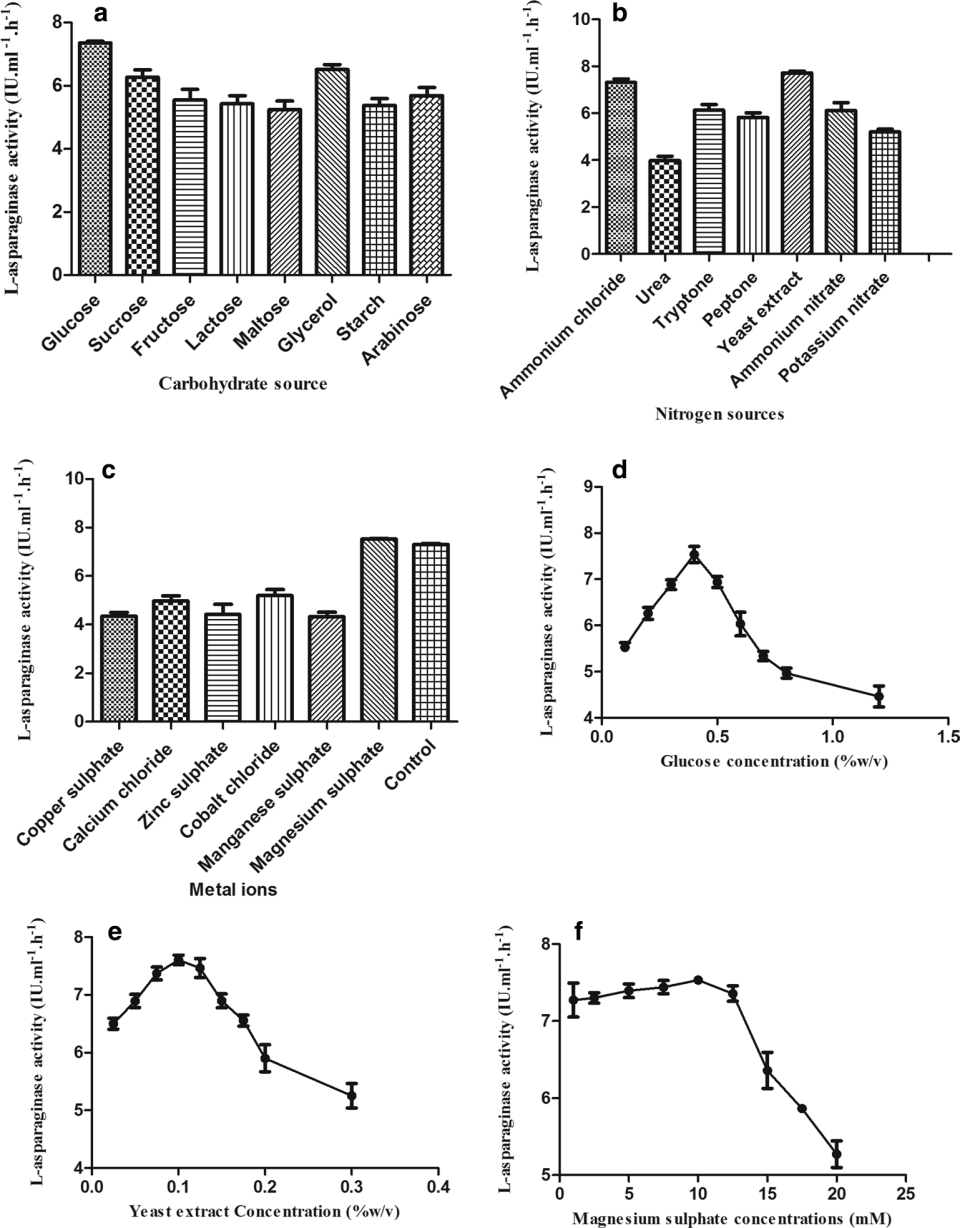
l-asparaginase production by *Stenotrophomonas maltophilia* EMCC2297 variant at various sources of **a** carbohydrate, **b** nitrogen, **c** metal salts and various concentrations of **d** dextrose, **e** yeast extract and **f** sulphate salt of magnesium. In all cases (**a**–**f**) p-values were < 0.0001 which indicates high significance of the correlation

By studying the influence of various carbohydrate sources which included maltose, glycerol, fructose, starch, dextrose, sucrose, lactose and arabinose, dextrose (at 0.5% w/v), yeast extract (at 0.1% w/v), magnesium (10 mM) were the best carbohydrate, nitrogen source, metal ions for maximum production of l-asparaginase, respectively (Fig. 6).

## Discussion

l-asparaginase has received considerable attention as a primary component in the treatment of acute lymphoblastic leukemia (ALL) (Sinha et al. 2013). Also acts as a biosensor to detect the amount of asparagine in leukemia and food industry (Batool et al. 2016). A complete identification of our microorganism was done by biochemical tests and confirmed by the 16S rRNA sequencing. The genotypic identification of bacteria is generally more accurate than the traditional identification on the basis of phenotypic characteristics. The 16S rRNA sequencing was found to be a phylogenetic tool also useful for bacterial detection and identification, it can better identify poorly described strains (Matsumoto and Sugano 2013). Extracellular enzymes have an advantage of being they don’t need cell breakage allowing their cost effective purification (Deokar et al. 2010; Joseph and Rajan 2011). Fortunately, our test isolate that used in the present study could produce l-asparaginase extracellularly (Type II l-asparaginase) at a level 470% more than its intracellular level. Bacterial l-asparaginases are defined by their intra or extra cellular localization (Michalska and Jaskolski 2006). Type I (cytosolic) binds to l-asparagine with low affinity, in contrast the extracellular type II (periplasmic) has a strong binding capacity and it has a characteristic oligomeric form. For more than 30 years, type II asparaginases (Campbell and Mashburn 1969), from *Escherichia coli* and *Erwinia chrysanthemi*, were used in the treatment of acute lymphoblastic leukemia and some other tumor types (Bodey et al. 1974; Boyse et al. 1967; Roberts et al. 1966; Lay et al. 1975). Thus, the extracellular l-asparaginase of the test isolate was our focus in the present study.

By testing the phylogeny and antigenicity for l-asparaginase of *Stenotrophomonas maltophilia*, it is obvious that l-asparaginases of *Stenotrophomonas* species (*maltophilia* and *bentonitica*) are clustered with two *Pseudomonas* species (*weihenstephanensis* and *lundensis*). This cluster shows different degree of relatedness to clusters and sub-clusters of other *Pseudomonas* species presented. l-asparaginases of *Pseudomonas fragi* and *Pseudomonas lundensis* are distantly related. The relationship between the plant type l-asparaginase and bacterial species ones is an important factor to allow the formation of complete profile for *Stenotrophomonas*l-asparaginase so a comparison between them was done, the results demonstrated the degree of closeness and relatedness of these enzyme sources. Although these enzyme sources could be seen in different clusters in the tree, the maximum obtained pairwise distance did not exceed 0.7175 which was recorded between *Pseudomonas lundensis* and *Pseudomonas fragi*l-asparaginases. Measuring the corresponding distances among bacterial species l-asparaginases and those of plant type ones showed clustering of *E. coli*l-asparaginase with that of *Erwinia chrysanthemi* with a pairwise distance of 0.750 and considerable relatedness of *Stenotrophomonas maltophilia*l-asparaginase with each of *E. coli* and *E. chrysanthemi*, pairwise distances 1.201 and 1.191, respectively. A distant relatedness was observed between the plant type l-asparaginase and those of *Stenotrophomonas maltophilia, E. coli* and *E. chrysanthemi* with pairwise distances of 2.233, 2.398 and 2.197, respectively. Analysis of the antigenic epitopes showed that the lowest antigenicity was obtained with *Stenotrophomonas maltophilia*l-asparaginase followed by that of *Erwinia chrysanthemi* while that of *E. coli* is the one of the highest number of antigenic regions. Cavanna et al. (1976) stated that depressive and toxic immune mediated effects were demonstrated in l-asparaginase from *E. coli* than from *E. carotovora*. This result validates the predicted results obtained for l-asparaginases of the three aforementioned strains using EMBOSS antigenic explorer^®^ (Barry et al. 2007). The medical application of l-asparaginases from *E. coli* and *Erwinia* is accompanied by partial immune mediated adverse effects of these enzymes. This necessitates the search for other microbial l-asparaginases with minimal immune mediated side effects. Lower antigenic epitopes predicted in l-asparaginase of *Stenotrophomonas maltophilia* suggests less adverse effects which could support further experimental analysis in vivo animal model for proving the potential introduction of *Stenotrophomonas maltophilia*l-asparaginase as a therapeutic candidate for medical application.

The aim of this study was not only increasing the production of l-asparaginase but also selecting the l-asparaginase that had industrially needed characteristics. The special characteristics of the enzymes exploited for their industrial applications and commercial interest were tested. The difference between the native and denatured structure of the enzymes limits their applications as the enzymes became fragile in nature. So the enzymes that show stability over pH ranges and temperature are highly needed in the industrial field. For medical applications thermal stability to temperature higher than this level (50 °C) are mostly not required. These results were the same as that reported by Mahajan et al. (2014) whose results showed that l-asparaginase produced from *B. licheniformis* was more robust than that produced from *E. coli* (Kumar et al. 2011). However, our results are in agreement with those reported by other authors (Elshafei et al. 2012). Regarding the optimum pH for l-asparaginase activity it was found that the maximum activity occurs at neutral pH (7) or alkaline pH (8 and 9). Maximum enzyme activity from *Bacillus* sp. and *Stenotrophomonas maltophilia* at neutral pH was reported by Abdel-Fattah and Olama (2002), El-Mched et al. (2015) and Maysa et al. (2010) while maximum activity at alkaline pH (8 and 9) for *Streptomyces halstedii*, *Penicillium brevicompactum*, *B. licheniformis* and *Streptomyces gulbargensis*l-asparaginases was reported by Amena et al. (2010), El-Sabbagh et al. (2013), Elshafei et al. (2012) and Mahajan et al. (2014). The optimum pH for activity obtained in our study was alkaline (8.6) that could be explained in the view of enzyme mechanistic action in terms of the aspartic acid and aspartate balance. At acidic pH aspartic acid had higher affinity at the enzyme active site, while at the alkaline pH aspartate had lower affinity to the active site which enabled the binding of asparagine to the enzyme. Also may be due to within acidic pH inhibition by competition for l-asparaginase is exerted by aspartic acid (El-Sabbagh et al. 2013). It was reported by Persson and Halle (2008) that the biological activity of proteins is essentially dependent on water molecules which attach to the surface and enter into the protein molecules. The water activity in biological system is affected by a various conditions such as extreme pH, temperature or high sodium chloride concentrations. Fortunately in our study the l-asparaginase produced by *Stenotrophomonas maltophilia* EMCC2297 exhibited increased activity by about 50% at half molar sodium chloride concentration. This increase in activity could be interpreted by the explanation reported by Han et al. (2014) who stated that salinity can render the structure of the enzyme more flexible in such a way that increases the enzyme activity. The same authors reported that negative charges are associated with halophilic enzymes and the presence of sodium chloride renders these enzymes more flexible with an increase in enzyme activity. The results reported in this study agreed those obtained by many researchers (Dash et al. 2016; Elshafei et al. 2012; Han et al. 2014; Shechtman 2013) who reported that sodium chloride increases enzyme activity. Regarding l-asparaginase activity produced by *Stenotrophomonas maltophilia* test isolate in relation to asparagine concentration, it was found that this relationship obeys the commonly exhibited by various enzymes. That is a proportional increase in enzyme activity occurs by increasing the substrate concentration to a certain level after which either plateau or a decrease in enzyme activity usually happens. Similar results were stated by El-Mched et al. (2015) and the absence of the increase of enzyme activity after certain substrate concentration may be due to the saturation of the enzyme catalytic active site. The results of the present study for *Stenotrophomonas maltophilia* EMCC2297 l-asparaginase and as reported by Abdelrazek et al. (2019) the chloride salts of either nickel or cobalt and the sulphate salts of either copper or ferrous were not required for l-asparaginase activity. Contrary to the results reported by^28^, the sulphate salt of zinc did not increase the l-asparaginase activity produced by the test isolate. Existence of metal ions reasonably activate the enzyme allowing its suitability for industrial application (Badoei-Dalfard 2016). Fortunately, the maximum activity for l-asparaginase of *Stenotrophomonas maltophilia* EMCC2297 was obtained at body temperature (37 °C) that can be important for its medical use to assure complete elimination of asparagine from cancer patient.

Strain improvement is an important part of development of microbial product. Arising of strains with high productivity can reduce costs. In the present study mutagenesis was conducted by gamma rays (physical mutagen). Gamma rays is the highest ionizing radiation as well as the most energetic, it causes mutation through breakage of single and double stranded DNA resulting structural changes or oxidation (Huma et al. 2012). Gamma irradiation was reported by many authors (Diep et al. 2017; Hoe et al. 2016; Huma et al. 2012; Hyster and Ward 2016) as an efficient physical mutagen for improving the enzyme production by different microbial strain.

Optimization of production conditions as well as production media components can help in increasing the enzyme production to many folds. So optimization through studying environmental conditions and statistical design of experiments by Response Surface Methodology (RSM) was performed, (RSM) is a statistical program applying mathematical calculations for developing a correlation between a target response and number of variables. The incubation temperature is an important environmental factor for l-asparaginase production by microorganisms, as it regulates the microbial growth and consequently the enzyme production (El-Hefnawy et al. 2015). In our study the maximum production was obtained at 37 °C either higher or lower temperature showed a decrease in enzyme production as a result of slowing down the microorganisms’ metabolic activity (El-Hefnawy et al. 2015). Many researchers reported similar results, where maximum productivity of l-asparaginase was obtained at 37 °C (Bahrani 2016; El-Hefnawy et al. 2015). While others recorded 39 °C as optimum temperature (Prakasham et al. 2007) or 30 °C (Jayaramu et al. 2010). The growth of *Stenotrophomonas maltophilia* at different pHs revealed that higher or lower initial pH value than the optimum showed decline in enzyme productivity as it may adversely affect the enzyme production or its activity or both. This agreed with several studies (Bahrani 2016; Kavitha and Vijayalakshmi 2010). However, other optimum pH values (6.5, 7.5, 6) for l-asparaginase production were reported by Jayaramu et al. (2010), Pradhan et al. (2013) and Prakasham et al. (2007). The incubation time required for maximum product formation differs according to the microbial strain applied (Maysa et al. 2010; Pradhan et al. 2013). On the other hand, studying the effect of the incubation showed steep wise decrease in enzyme productivity thereafter the optimum time.

The decrease in enzyme productivity after its maximum production level mostly attributed to the enzyme degradation by proteolytic enzyme of the producing organism or production of inhibitors upon depletion of the culture media components. Enzyme formation and production at short incubation periods is preferable for commercial enzyme production as its cost effectiveness and because of the decomposition liability of the produced enzyme is minimized (El-Hefnawy et al. 2015). Various incubation periods were recorded for maximal l-asparaginase production by different microorganisms, 48 h for *Emericella nidulans* and *Stenotrophomonas maltophilia* (Jayaramu et al. 2010; Kavitha and Vijayalakshmi 2010) and 72 h for *Streptomyces tendae* and *Penicillium oxalicum* (El-Hefnawy et al. 2015; Kavitha and Vijayalakshmi 2010) and 120 h for *Fusarium* spp. (Murali 2011). Regarding the agitation rate, the reason for this result is that agitation could affect the physiological status of the microorganism and consequently its production capability for certain enzyme or metabolite. Although increased agitation could increase nutrition and oxygen availability (Sooch and Kauldhar 2013) and increase nutrient absorption by the microorganisms as well (Pansuriya and Singhal 2011), it might decrease the product formation which could be attributed to the shear stress exerted on the bacterial cells especially at higher agitation rates (Sooch and Kauldhar 2013). Other published researches reported higher production at 220 rpm (Bahrani 2016) and 150 rpm (Pradhan et al. 2013).

After applying the RSM to the four tested parameters, the model showed large model F-value which could occur at a low chance (0.01%) and this is attributed to noise. A very low probability value (P model > F) = 0.0001 of Fisher’s F-test gives an evidence that the model is of high significance. The model coefficient regression (R^2^) was calculated to indicate the model fitness, its value indicating that the design could clarify the variability by 94.75%. Different statistical criteria have to be checked for validation of the applied model. For example a determination coefficient of high value refers to that the model cannot explain the total variation by a percentage not exceeding 5.25%. The lack of fit F-value of 6.97 and P-value of 0.1319 indicate that lack of fit is non-significant as compared to the pure error as it contrasts the residual error with the “pure error” from replicated design points and that model exhibit excellent fit. The value of the “Adjusted R-Squared” refers that the model can clarify the variability by 88.6% if the sample was a group of the parameters other than the studied ones, its value is always less than the (R^2^) (Frost 2013). The “Predicted R-Squared” is in considerable compliance (a difference not more than the recommended 0.3 value with the “Adjusted R-Squared”) (Frost 2013). This means that the model is of high prediction ability and could explain 70% of observations in addition to the fed in data that can be successfully anticipated by the model (Frost 2013). “Adequate precision” is expressed in terms of signal to noise ratio. The cut off value of signal to noise ratio for the model is 4 and values greater than this value indicates sufficient and satisfactory signal to noise ratio. Therefore the design space can be navigated by the applied model. The overall consistency of measure determines the model reliability. It is measured by the coefficient of variation which is equal to the standard deviation to the mean ratio. The reliability and precision of the model are attained at lower CV values, its value implies high reliability and excellent precision (Shechtman 2013). For the CV to be significant F-value magnitude should be large and P-value magnitude should be small (Adinarayana and Ellaiah 2002).

Although dextrose catabolic regression its incorporation could enhance the enzymes/metabolites production by various microorganisms. In the present study dextrose proved to have a positive effect on l-asparaginase biosynthesis by the test organism as it increased the production level of this enzyme. Other authors also reported that dextrose increases the production of l-asparaginase as in case of *Aeromonas* sp., *Aspergillus terreus* (Amena et al. 2010; Doriya and Kumar 2016; El-Hefnawy et al. 2015; Gurunathan and Sahadevan 2011; Varalakshmi and Raju 2013). However, other carbohydrate sources were reported for their enhancing effect on l-asparaginase biosynthesis as in case of sucrose and sorbitol for *Streptomyces tendae* (Kavitha and Vijayalakshmi 2010) and lactose in case of *E. coli* (Bahrani 2016). Stimulation of the production occurred at low glucose concentrations while inhibition happened at high concentrations which may be due to the catabolic repression effect of glucose. The same results were reported by other workers (El-Hefnawy et al. 2015; Gurunathan and Sahadevan 2011). Enzymes productions are highly dependent on the used sources of nitrogen, since these sources affect nucleic acid production and cell wall and protein biosynthesis. In our study yeast extract proved to be the best nitrogen source for l-asparaginase production and this is in agreement with that reported by many researchers (Bahrani 2016; El-Hefnawy et al. 2015; Kavitha and Vijayalakshmi 2010) while ammonium sulphate was the best in others (Varalakshmi and Raju 2013). The maximum production was occurred at 0.1% yeast extract concentration and it was 0.5% and 2% in different studies (Amena et al. 2010; El-Hefnawy et al. 2015; Kavitha and Vijayalakshmi 2010). The increase in l-asparaginase production by magnesium, also recorded in Megavarnam and Janakiraman (2015) and Varalakshmi and Raju (2013). In our study, the maximum l-asparaginase production was obtained at 10 mM magnesium sulphate followed by a remarkable decrease in production of the enzyme by increasing the concentration.

In conclusion, the recovered *Stenotrophomonas maltophilia* EMCC2297 soil isolate produces l-asparaginase with promising characteristics that can support further work and investigations for its introduction as a candidate for medical and pharmaceutical applications, as it showed lower predicted antigenicity compared to the commercially available used enzymes (Elspar and Erwinaze).

From the present study we can conclude that soil represents an inestimable source of useful microorganisms, among which l-asparaginase producing bacteria. l-asparaginase has a crucial medical application as anticancer agent. The soil recovered isolate, *Stenotrophomonas maltophilia,* proved to be a promising source for l-asparaginase that can be used medically as it showed lower predicted antigenicity as compared to the commercial available ones (Elspar and Erwinaze). Production conditions for maximum l-asparaginase production were determined in this study and could be applied for commercial exploitation.

## Supplementary information

**Additional file 1: Table S1.** Query coverage, E value, percent identity and accession numbers of amino acid sequences of l-asparaginases for the tested bacterial species as revealed by NCBI databases. **Table S2.** Pairwise distances among l-asparaginases of bacterial species presented in the phylogenetic tree shown in Fig. 1. **Table S3.** Pairwise distances among bacterial l-asparaginases of *Stenotrophomonas maltophilia*, *E. coli* and *Erwinia chrysanthemi* and the plant l-asparaginase of *Medicago truncatula* presented in the phylogenetic tree shown in Fig. S1. **Table S4.** ANOVA of the quadratic model for the process parameters optimization of l-asparaginase productivity by *Stenotrophomonas maltophilia* EMCC2297 mutant using Box–Behnken central composite design. **Table S5.** Levels of reaction conditions of process parameters as independent variables studied in RSM experimental design for optimization of l-asparaginase production by the selected test mutant. **Table S6.** Experiments that were deduced by the RSM experimental design and tested for l-asparaginase production by the test mutant. **Fig. S1.** Molecular Phylogenetic analysis by Maximum Likelihood method of *Stenotrophomonas maltophilia*l-asparaginase when blasted against amino acid sequences of the FDA approved l-asparaginases of *E. coli* and *Erwinia chrysanthemi* and the plant type l-asparaginase of *Medicago truncatula*.

## Data Availability

Please contact author for data request.
