# Supplementary material for: Production, characterization and bioinformatics analysis of l-asparaginase from a new Stenotrophomonas maltophilia EMCC2297 soil isolate
Source: AMB Express. 2020 Apr 15;10:71. doi: 10.1186/s13568-020-01005-7 (PMC7158977; doi:10.1186/s13568-020-01005-7)

**AMB Express**

**Production, characterization and bioinformatics analysis of L-asparaginase from a new *Stenotrophomonas maltophilia* EMCC2297 soil isolate**

**Nada A.Abdelrazek^1^, Walid F. Elkhatib^2,3^,** **Marwa M. Raafat^1^, Mohammad M. Aboulwafa^2^*,**

^1^Department of Microbiology and Immunology, Faculty of Pharmaceutical sciences and Pharmaceutical industries, Future University, Cairo, Egypt

^2^Department of Microbiology and immunology, Faculty of Pharmacy Ain Shams University, Cairo, Egypt

^2^Department of Microbiology and Immunology, School of Pharmacy & Pharmaceutical Industries, Badr University in Cairo (BUC), Entertainment Area, Badr City, Cairo, Egypt

*** Corresponding author:**  Mohammad M. Aboulwafa

Address: Department of Microbiology and Immunology, Faculty of Pharmacy, Ain Shams University, Al Khalifa Al Maamoun St., Abbassia, Cairo, Egypt.

E-mail: maboulwafa@yahoo.com Tel: (202)23892562

Mobile: (02)01002350371 Fax: (202)24051107

Postal code: 11517

ORCID: 0000-0002-0828-1420

**Prof. Dr. Walid F. Elkhatib**

Postal address: Department of Microbiology & Immunology, Faculty of Pharmacy, Ain Shams University, African Union Organization St. Abbassia, Cairo 11566, Egypt

Tel: +202-24051120, Fax: +202-24051107

Email: [walid-elkhatib@pharma.asu.edu.eg](mailto:walid-elkhatib@pharma.asu.edu.eg)

ORCID: 0000-0001-5815-3200

**Dr. Marwa M. Raafat**

Department of Microbiology & Immunology, Faculty of Pharmaceutical Sciences and Pharmaceutical Industries, Future University in Egypt.

E-mail: [Marwa.Mahmoud@fue.edu.eg](mailto:Marwa.Mahmoud@fue.edu.eg); [marwrft@yahoo.com](mailto:marwrft@yahoo.com)

ORCID: 0000-0001-5614-5127

**Nada A. Abdelrazek**

Department of Microbiology & Immunology, Faculty of Pharmaceutical Sciences and Pharmaceutical Industries, Future University in Egypt.

E-mail: [Nada.Anwar@fue.edu.eg](mailto:Nada.Anwar@fue.edu.eg); n_s1986@hotmail.com

ORCID: 0000-0001-8082-2298

**Table S1 Query coverage, E value, percent identity and accession numbers of amino acid sequences of L-asparaginases for the tested bacterial species as revealed by NCBI databases**

| Bacterial species | [Query Cover](https://blast.ncbi.nlm.nih.gov/Blast.cgi?CMD=Get&ADV_VIEW=yes&ADV_VIEW=on&ALIGNDB_BATCH_ID=536532542&ALIGNDB_CGI_HOST=blast.be-md.ncbi.nlm.nih.gov&ALIGNDB_CGI_PATH=/ALIGNDB/alndb_asn.cgi&ALIGNDB_MASTER_ALIAS=SD_ALIGNDB_MASTER&ALIGNDB_MAX_ROWS=500&ALIGNDB_ORDER_CLAUSE=seq_evalue%20asc,aln_id%20asc&ALIGNDB_WHERE_CLAUSE=seq_evalue%20is%20not%20null&ALIGNMENTS=250&ALIGNMENT_VIEW=Pairwise&CDD_RID=data_cache_seq:WP_102789458.1&CDD_SEARCH_STATE=4&DATABASE_SORT=0&DESCRIPTIONS=500&DYNAMIC_FORMAT=on&FIRST_QUERY_NUM=0&FORMAT_NUM_ORG=1&FORMAT_OBJECT=Alignment&FORMAT_PAGE_TARGET=&FORMAT_TYPE=HTML&GET_SEQUENCE=yes&I_THRESH=&LINE_LENGTH=60&MASK_CHAR=2&MASK_COLOR=1&NEW_VIEW=yes&NUM_OVERVIEW=500&PAGE=Proteins&QUERY_INDEX=0&QUERY_NUMBER=0&RESULTS_PAGE_TARGET=&RID=5UWBY9RY016&SHOW_LINKOUT=yes&SHOW_OVERVIEW=yes&STEP_NUMBER=&USE_ALIGNDB=true&WORD_SIZE=6&ADV_VIEW=on&DISPLAY_SORT=4&HSP_SORT=0) | [E value](https://blast.ncbi.nlm.nih.gov/Blast.cgi?CMD=Get&ADV_VIEW=yes&ADV_VIEW=on&ALIGNDB_BATCH_ID=536532542&ALIGNDB_CGI_HOST=blast.be-md.ncbi.nlm.nih.gov&ALIGNDB_CGI_PATH=/ALIGNDB/alndb_asn.cgi&ALIGNDB_MASTER_ALIAS=SD_ALIGNDB_MASTER&ALIGNDB_MAX_ROWS=500&ALIGNDB_ORDER_CLAUSE=seq_evalue%20asc,aln_id%20asc&ALIGNDB_WHERE_CLAUSE=seq_evalue%20is%20not%20null&ALIGNMENTS=250&ALIGNMENT_VIEW=Pairwise&CDD_RID=data_cache_seq:WP_102789458.1&CDD_SEARCH_STATE=4&DATABASE_SORT=0&DESCRIPTIONS=500&DYNAMIC_FORMAT=on&FIRST_QUERY_NUM=0&FORMAT_NUM_ORG=1&FORMAT_OBJECT=Alignment&FORMAT_PAGE_TARGET=&FORMAT_TYPE=HTML&GET_SEQUENCE=yes&I_THRESH=&LINE_LENGTH=60&MASK_CHAR=2&MASK_COLOR=1&NEW_VIEW=yes&NUM_OVERVIEW=500&PAGE=Proteins&QUERY_INDEX=0&QUERY_NUMBER=0&RESULTS_PAGE_TARGET=&RID=5UWBY9RY016&SHOW_LINKOUT=yes&SHOW_OVERVIEW=yes&STEP_NUMBER=&USE_ALIGNDB=true&WORD_SIZE=6&ADV_VIEW=on&DISPLAY_SORT=0&HSP_SORT=0) | [Percent Ident](https://blast.ncbi.nlm.nih.gov/Blast.cgi?CMD=Get&ADV_VIEW=yes&ADV_VIEW=on&ALIGNDB_BATCH_ID=536532542&ALIGNDB_CGI_HOST=blast.be-md.ncbi.nlm.nih.gov&ALIGNDB_CGI_PATH=/ALIGNDB/alndb_asn.cgi&ALIGNDB_MASTER_ALIAS=SD_ALIGNDB_MASTER&ALIGNDB_MAX_ROWS=500&ALIGNDB_ORDER_CLAUSE=seq_evalue%20asc,aln_id%20asc&ALIGNDB_WHERE_CLAUSE=seq_evalue%20is%20not%20null&ALIGNMENTS=250&ALIGNMENT_VIEW=Pairwise&CDD_RID=data_cache_seq:WP_102789458.1&CDD_SEARCH_STATE=4&DATABASE_SORT=0&DESCRIPTIONS=500&DYNAMIC_FORMAT=on&FIRST_QUERY_NUM=0&FORMAT_NUM_ORG=1&FORMAT_OBJECT=Alignment&FORMAT_PAGE_TARGET=&FORMAT_TYPE=HTML&GET_SEQUENCE=yes&I_THRESH=&LINE_LENGTH=60&MASK_CHAR=2&MASK_COLOR=1&NEW_VIEW=yes&NUM_OVERVIEW=500&PAGE=Proteins&QUERY_INDEX=0&QUERY_NUMBER=0&RESULTS_PAGE_TARGET=&RID=5UWBY9RY016&SHOW_LINKOUT=yes&SHOW_OVERVIEW=yes&STEP_NUMBER=&USE_ALIGNDB=true&WORD_SIZE=6&ADV_VIEW=on&DISPLAY_SORT=3&HSP_SORT=3)ity | Accession number |
| --- | --- | --- | --- | --- |
| *Pseudomonas fuscovaginae* | 97% | 2e-121 | 57.32% | WP_054059885.1 |
| [asparaginase *Pseudomonas fragi*](https://blast.ncbi.nlm.nih.gov/Blast.cgi#alnHdr_WP_016779508) | 95% | 1e-124 | 56.07% | WP_016779508.1 |
| *Stenotrophomonas maltophilia* | 100% | 0.0 | 97.91% | WP_088435380.1 |
| *Stenotrophomonas bentonitica* | 100% | 0.0 | 100.00% | WP_102789458.1 |
| *Pseudomonas weihenstephanensis* | 97% | 2e-145 | 65.24% | WP_048401277.1 |
| *Pseudomonas lundensis* | 96% | 3e-138 | 63.47% | WP_094993066.1 |
| *Pseudomonas lundensis* (2) | 96% | 1e-137 | 62.85% | WP_070413770.1 |
| *Pseudomonas reinekei* | 96% | 4e-138 | 61.30% | WP_075945639.1 |
| *Pseudomonas mohnii* | 97% | 8e-137 | 59.69% | WP_090464963.1 |
| *Pseudomonas putida* | 96% | 5e-141 | 61.73% | WP_082240437.1 |
| *Pseudomonas umsongensis* | 97% | 3e-140 | 60.92% | WP_083348263.1 |
| *Pseudomonas jessenii* | 96% | 8e-135 | 58.70% | WP_110660697.1 |
| *Pseudomonas laurylsulfatiphila* | 97% | 2e-137 | 59.38% | WP_104449371.1 |
| *Pseudomonas fluorescens* | 96% | 8e-141 | 60.49% | WP_150702963.1 |
| *Pseudomonas migulae* | 97% | 3e-136 | 58.77% | WP_084320461.1 |
| *Pseudomonas syringae* | 96% | 2e-137 | 60.06% | WP_052966301.1 |
| *Pseudomonas frederiksbergensis* | 97% | 2e-141 | 60.92% | WP_074880127.1 |
| *Pseudomonas agarici* | 97% | 3e-138 | 60.24% | WP_017132649.1 |
| *[Pseudomonas prosekii* | 97% | 5e-138 | 61.54% | WP_109502501.1 |

**Table S2: Pairwise distances among L-asparaginases of bacterial species presented in the phylogenetic tree shown in Figure 1.**

|  |  | 1 | 2 | 3 | 4 | 5 | 6 | 7 | 8 | 9 | 10 | 11 | 12 | 13 | 14 | 15 | 16 | 17 | 18 | 19 |
| --- | --- | --- | --- | --- | --- | --- | --- | --- | --- | --- | --- | --- | --- | --- | --- | --- | --- | --- | --- | --- |
| 1 | asparaginase_*Stenotrophomonas_maltophilia.* |  |  |  |  |  |  |  |  |  |  |  |  |  |  |  |  |  |  |  |
| 2 | asparaginase_*Stenotrophomonas_bentonitica.* | 0.0211 |  |  |  |  |  |  |  |  |  |  |  |  |  |  |  |  |  |  |
| 3 | asparaginase_*Pseudomonas_weihenstephanensis.* | 0.4425 | 0.4331 |  |  |  |  |  |  |  |  |  |  |  |  |  |  |  |  |  |
| 4 | asparaginase_*Pseudomonas_frederiksbergensis.* | 0.4986 | 0.4986 | 0.4398 |  |  |  |  |  |  |  |  |  |  |  |  |  |  |  |  |
| 5 | L-asparaginase_*Pseudomonas_putida*. | 0.5088 | 0.5038 | 0.5008 | 0.2224 |  |  |  |  |  |  |  |  |  |  |  |  |  |  |  |
| 6 | asparaginase_*Pseudomonas_umsongensis.* | 0.5189 | 0.5139 | 0.5159 | 0.2186 | 0.1288 |  |  |  |  |  |  |  |  |  |  |  |  |  |  |
| 7 | asparaginase_*Pseudomonas_lundensis.* | 0.4773 | 0.4821 | 0.3102 | 0.4688 | 0.5189 | 0.5343 |  |  |  |  |  |  |  |  |  |  |  |  |  |
| 8 | asparaginase_*Pseudomonas_fluorescens.* | 0.5291 | 0.5240 | 0.5008 | 0.1922 | 0.2376 | 0.2376 | 0.5038 |  |  |  |  |  |  |  |  |  |  |  |  |
| 9 | asparaginase_*Pseudomonas_agarici.* | 0.5160 | 0.5108 | 0.4511 | 0.1744 | 0.2347 | 0.2270 | 0.4756 | 0.2193 |  |  |  |  |  |  |  |  |  |  |  |
| 10 | asparaginase_*Pseudomonas_prosekii.* | 0.4905 | 0.4855 | 0.4463 | 0.2003 | 0.2426 | 0.2504 | 0.4657 | 0.2347 | 0.1455 |  |  |  |  |  |  |  |  |  |  |
| 11 | asparaginase_*Pseudomonas_reinekei.* | 0.5006 | 0.4956 | 0.4608 | 0.2079 | 0.2270 | 0.2193 | 0.5006 | 0.2117 | 0.2086 | 0.2239 |  |  |  |  |  |  |  |  |  |
| 12 | asparaginase_*Pseudomonas_lundensis*.(2) | 0.4878 | 0.4829 | 0.3755 | 0.4639 | 0.4977 | 0.5028 | 0.1577 | 0.4730 | 0.4956 | 0.4657 | 0.5057 |  |  |  |  |  |  |  |  |
| 13 | asparaginase_*Pseudomonas_syringae.* | 0.5190 | 0.5190 | 0.4688 | 0.1738 | 0.2224 | 0.2186 | 0.4936 | 0.1701 | 0.1966 | 0.2003 | 0.1928 | 0.4737 |  |  |  |  |  |  |  |
| 14 | asparaginase_*Pseudomonas_mohnii.* | 0.5273 | 0.5221 | 0.4572 | 0.2147 | 0.2526 | 0.2526 | 0.5017 | 0.2178 | 0.2504 | 0.2624 | 0.1891 | 0.5068 | 0.2339 |  |  |  |  |  |  |
| 15 | asparaginase_*Pseudomonas_jessenii.* | 0.5368 | 0.5421 | 0.5160 | 0.1935 | 0.1891 | 0.2117 | 0.5263 | 0.2347 | 0.2443 | 0.2642 | 0.2325 | 0.5160 | 0.2086 | 0.2704 |  |  |  |  |  |
| 16 | asparaginase_*Pseudomonas_laurylsulfatovorans.* | 0.5377 | 0.5325 | 0.5068 | 0.2034 | 0.1768 | 0.1952 | 0.5170 | 0.2293 | 0.2347 | 0.2386 | 0.2231 | 0.5068 | 0.2034 | 0.2645 | 0.0473 |  |  |  |  |
| 17 | asparaginase_*Pseudomonas_migulae.* | 0.5399 | 0.5346 | 0.4542 | 0.2186 | 0.2417 | 0.2735 | 0.4886 | 0.2262 | 0.2155 | 0.2584 | 0.2465 | 0.4836 | 0.1922 | 0.2816 | 0.2593 | 0.2417 |  |  |  |
| 18 | asparaginase_*Pseudomonas_fuscovaginae.* | 0.5566 | 0.5566 | 0.5149 | 0.4116 | 0.4603 | 0.4603 | 0.5835 | 0.4412 | 0.4415 | 0.4225 | 0.4085 | 0.5835 | 0.4398 | 0.4572 | 0.4367 | 0.4334 | 0.4590 |  |  |
| 19 | asparaginase_*Pseudomonas_fragi*. | 0.6434 | 0.6264 | 0.6516 | 0.5294 | 0.5566 | 0.5498 | 0.7175 | 0.5446 | 0.5368 | 0.5211 | 0.5211 | 0.6781 | 0.5346 | 0.5970 | 0.4956 | 0.4916 | 0.5612 | 0.5566 |  |

**Table S3: Pairwise distances among bacterial L-asparaginases of *Stenotrophomonas maltophilia*, *E. coli* and *Erwinia chrysanthemi* and the plant L-asparaginase of *Medicago truncatula* presented in the phylogenetic tree shown in Figure S1**

|  |  | 1 | 2 | 3 |
| --- | --- | --- | --- | --- |
| 1 | ansB *Escherichia coli* B354 |  |  |  |
| 2 | L-asparaginase *Dickeya* (*Erwinia*) *chrysanthemi* | 0.750 |  |  |
| 3 | L-asparaginase *Stenotrophomonas maltophilia* | 1.201 | 1.191 |  |
| 4 | Isoaspartyl peptidase/L-asparaginase *Medicago truncatula* | 2.398 | 2.197 | 2.233 |

**Table S4: ANOVA of the quadratic model for the process parameters optimization of** **L-asparaginase productivity by *Stenotrophomonas maltophilia*** EMCC2297 **mutant using Box-Behnken central composite design**

| **Source** | **SS** | **DF** | **MS** | **F-value** | **p-value** | **Comments** |
| --- | --- | --- | --- | --- | --- | --- |
| **Model** | 0.23 | 14 | 0.016 | 15.47 | <0.0001 | Significant |
| **Residual** | 0.013 | 12 | 1.045E-003 |  |  |  |
| **Lack of Fit** | 0.012 | 10 | 1.219E-003 | 6.97 | 0.1319 | not significant |
| **Total Regression** | 0.24 | 26 |  |  |  |  |
| **Analysis reliability parameters** | | | | | | |
| **R-Squared** | 0.9473 |  | | | | |
| **Adjusted R-Squared** | 0.8863 |  |  |  |  |  |
| **Predicted R-Squared** | 0.7028 |  |  |  |  |  |
| **Adequate Precision** | 13.564 |  |  |  |  |  |

*Where, SS, Sum of Squares; DF, Degrees of Freedom; MS, Mean of Squares.

**Table S5: Levels of reaction conditions of process parameters as independent variables studied in RSM experimental design for optimization of L-asparaginase production by the selected test mutant.**

| **Test variable** | **Variable code** | **Variables levels** |
| --- | --- | --- |
| **Incubation temperature (°C)** | **A** | **-1 0 +1** |
|  |  | **35 37.5 40** |
| **Initial pH** | **B** | **6 7 8** |
| **Incubation time (h)** | **C** | **18 33 48** |
| **Agitation (RPM)** | **D** | **150 175 200** |

**Table S6: Experiments that were deduced by the RSM experimental design and tested for L-asparaginase production by the test mutant.**

| **Experiment** | **Temperature (°C)** | | **pH** | | **Time (h)** | | **Agitation (rpm)** | |
| --- | --- | --- | --- | --- | --- | --- | --- | --- |
|  | **Value** | **Level code** | **Value** | **Level code** | **Value** | **Level code** | **Value** | **Level code** |
| 1 | 40 | 1 | 7 | 0 | 48 | 1 | 175 | 0 |
| 2 | 37.5 | 0 | 8 | 1 | 33 | 0 | 200 | 1 |
| 3 | 37.5 | 0 | 7 | 0 | 48 | 1 | 200 | 1 |
| 4 | 40 | 1 | 8 | 1 | 33 | 0 | 175 | 0 |
| 5 | 37.5 | 0 | 8 | 1 | 48 | 1 | 175 | 0 |
| 6 | 37.5 | 0 | 8 | 1 | 18 | -1 | 175 | 0 |
| 7 | 35 | -1 | 7 | 0 | 33 | 0 | 150 | -1 |
| 8 | 37.5 | 0 | 7 | 0 | 33 | 0 | 175 | 0 |
| 9 | 37.5 | 0 | 7 | 0 | 18 | -1 | 200 | 1 |
| 10 | 35 | -1 | 6 | -1 | 33 | 0 | 175 | 0 |
| 11 | 35 | -1 | 8 | 1 | 33 | 0 | 175 | 0 |
| 12 | 40 | 1 | 7 | 0 | 33 | 0 | 200 | 1 |
| 13 | 37.5 | 0 | 7 | 0 | 48 | 1 | 150 | -1 |
| 14 | 35 | -1 | 7 | 0 | 48 | 1 | 175 | 0 |
| 15 | 37.5 | 0 | 6 | -1 | 48 | 1 | 175 | 0 |
| 16 | 37.5 | 0 | 7 | 0 | 18 | -1 | 150 | -1 |
| 17 | 37.5 | 0 | 8 | 1 | 33 | 0 | 150 | -1 |
| 18 | 40 | 1 | 7 | 0 | 18 | -1 | 175 | 0 |
| 19 | 40 | 1 | 7 | 0 | 33 | 0 | 150 | -1 |
| 20 | 37.5 | 0 | 6 | -1 | 33 | 0 | 150 | -1 |
| 21 | 37.5 | 0 | 7 | 0 | 33 | 0 | 175 | 0 |
| 22 | 35 | -1 | 7 | 0 | 18 | -1 | 175 | 0 |
| 23 | 40 | 1 | 6 | -1 | 33 | 0 | 175 | 0 |
| 24 | 37.5 | 0 | 6 | -1 | 18 | -1 | 175 | 0 |
| 25 | 35 | -1 | 7 | 0 | 33 | 0 | 200 | 1 |
| 26 | 37.5 | 0 | 7 | 0 | 33 | 0 | 175 | 0 |
| 27 | 37.5 | 0 | 6 | -1 | 33 | 0 | 200 | 1 |


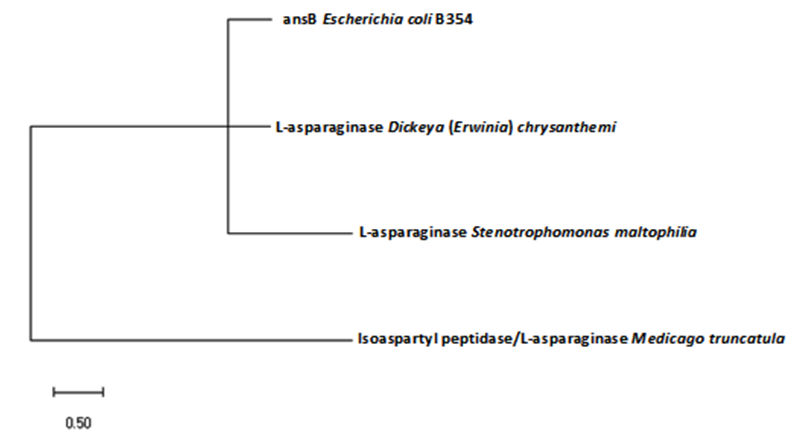


**Fig. S1** Molecular Phylogenetic analysis by Maximum Likelihood method of *Stenotrophomonas maltophilia* L- asparaginase when blasted against amino acid sequences of the FDA approved L- asparaginases of *E. coli* and *Erwinia chrysanthemi* and the plant type L-asparaginase of *Medicago truncatula*


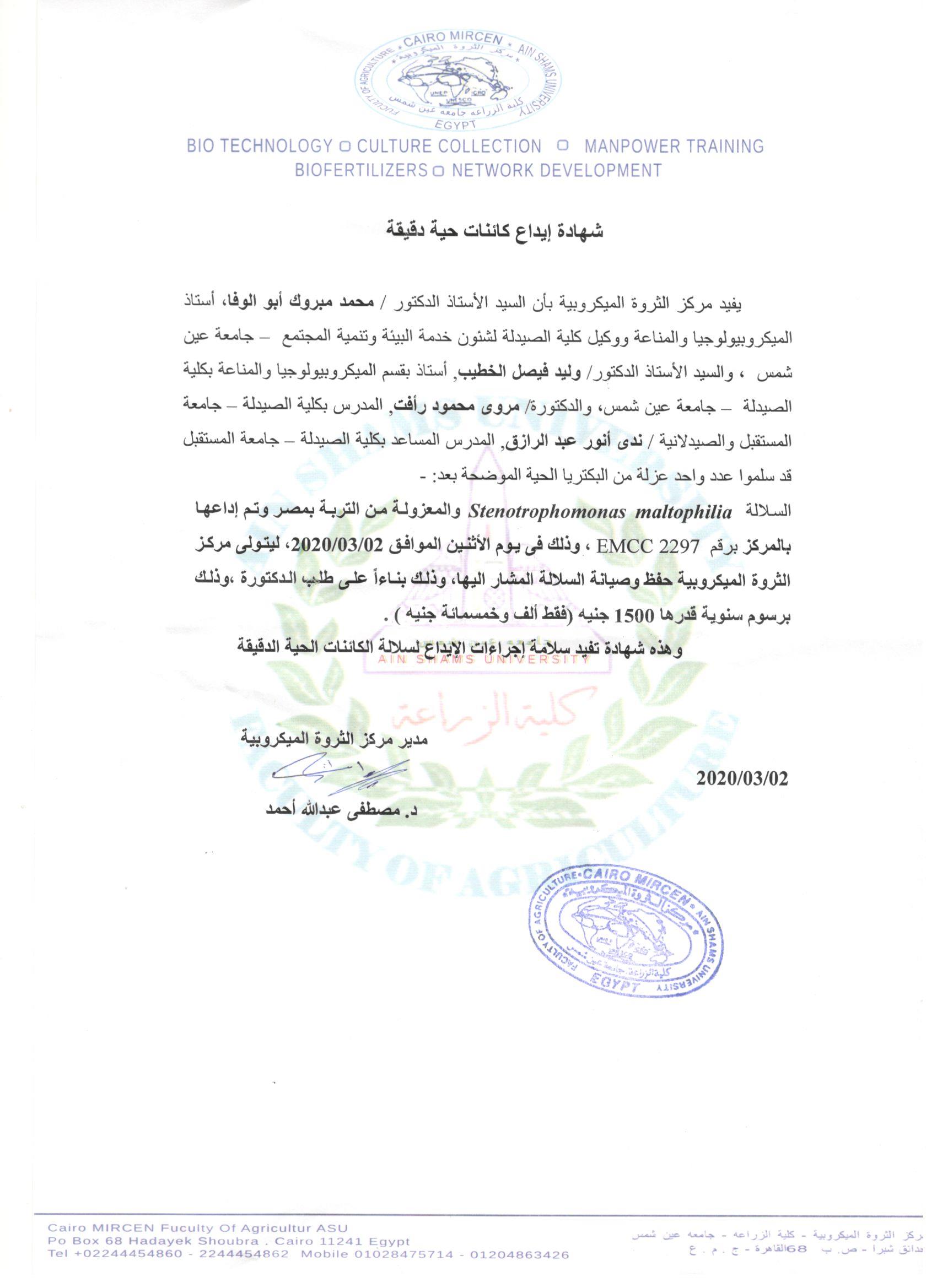

Supplement: Supplementary file 1 — Additional file 1: Table S1. Query coverage, E value, percent identity and accession numbers of amino acid sequences of l-asparaginases for the tested bacterial species as revealed by NCBI databases. Table S2. Pairwise distances among l-asparaginases of bacterial species presented in the phylogenetic tree shown in Fig. 1. Table S3. Pairwise distances among bacterial l-asparaginases of Stenotrophomonas maltophilia, E. coli and Erwinia chrysanthemi and the plant l-asparaginase of Medicago truncatula presented in the phylogenetic tree shown in Fig. S1. Table S4. ANOVA of the quadratic model for the process parameters optimization of l-asparaginase productivity by Stenotrophomonas maltophilia EMCC2297 mutant using Box–Behnken central composite design. Table S5. Levels of reaction conditions of process parameters as independent variables studied in RSM experimental design for optimization of l-asparaginase production by the selected test mutant. Table S6. Experiments that were deduced by the RSM experimental design and tested for l-asparaginase production by the test mutant. Fig. S1. Molecular Phylogenetic analysis by Maximum Likelihood method of Stenotrophomonas maltophilial-asparaginase when blasted against amino acid sequences of the FDA approved l-asparaginases of E. coli and Erwinia chrysanthemi and the plant type l-asparaginase of Medicago truncatula. [file 13568_2020_1005_MOESM1_ESM.docx]
